# Pre-treatment risk predictors of valproic acid-induced dyslipidemia in pediatric patients with epilepsy

**DOI:** 10.3389/fphar.2024.1349043

**Published:** 2024-04-02

**Authors:** Tiantian Liang, Chenquan Lin, Hong Ning, Fuli Qin, Bikui Zhang, Yichang Zhao, Ting Cao, Shimeng Jiao, Hui Chen, Yifang He, Hualin Cai

**Affiliations:** ^1^ Department of Pharmacy, The Second Xiangya Hospital of Central South University, Institute of Clinical Pharmacy, Central South University, Changsha, China; ^2^ Department of Pharmacy, Mianyang Central Hospital, School of Medicine, University of Electronic Science and Technology of China, Mianyang, China; ^3^ Institute of Clinical Pharmacy, Central South University, Changsha, China; ^4^ Department of Pharmacy, The First Affiliated Hospital of Guangxi Medical University, Nanning, China; ^5^ International Research Center for Precision Medicine, Transformative Technology and Software Services, Hunan, China

**Keywords:** valproic acid, dyslipidemia, epilepsy, pediatrics, risk predictors

## Abstract

**Background:** Valproic acid (VPA) stands as one of the most frequently prescribed medications in children with newly diagnosed epilepsy. Despite its infrequent adverse effects within therapeutic range, prolonged VPA usage may result in metabolic disturbances including insulin resistance and dyslipidemia. These metabolic dysregulations in childhood are notably linked to heightened cardiovascular risk in adulthood. Therefore, identification and effective management of dyslipidemia in children hold paramount significance.

**Methods:** In this retrospective cohort study, we explored the potential associations between physiological factors, medication situation, biochemical parameters before the first dose of VPA (baseline) and VPA-induced dyslipidemia (VID) in pediatric patients. Binary logistic regression was utilized to construct a predictive model for blood lipid disorders, aiming to identify independent pre-treatment risk factors. Additionally, The Receiver Operating Characteristic (ROC) curve was used to evaluate the performance of the model.

**Results:** Through binary logistic regression analysis, we identified for the first time that direct bilirubin (DBIL) (odds ratios (OR) = 0.511, *p* = 0.01), duration of medication (OR = 0.357, *p* = 0.009), serum albumin (ALB) (OR = 0.913, *p* = 0.043), BMI (OR = 1.140, *p* = 0.045), and aspartate aminotransferase (AST) (OR = 1.038, *p* = 0.026) at baseline were independent risk factors for VID in pediatric patients with epilepsy. Notably, the predictive ability of DBIL (AUC = 0.690, *p* < 0.0001) surpassed that of other individual factors. Furthermore, when combined into a predictive model, incorporating all five risk factors, the predictive capacity significantly increased (AUC = 0.777, *p* < 0.0001), enabling the forecast of 77.7% of dyslipidemia events.

**Conclusion:** DBIL emerges as the most potent predictor, and in conjunction with the other four factors, can effectively forecast VID in pediatric patients with epilepsy. This insight can guide the formulation of individualized strategies for the clinical administration of VPA in children.

## 1 Introduction

Epilepsy, a chronic brain disorder characterized by abnormal neuronal discharges, poses a common neurological challenge in children. Though its onset is often subtle, neonatal seizures related to epilepsy can be detrimental to the developing brain (Pack, 2019). According to epidemiological data, the incidence of epilepsy in children ranges from 33.3 to 82 cases per 100000 individuals annually (Fine and Wirrell, 2020), which is higher than that in adults. This condition can inflict significant neurological harm in children, leading to obstacles in independent learning, delayed brain development, intellectual impairment, and substantial impacts on growth, development, and psychological wellbeing (Beghi, 2020; Choi et al., 2022). Currently, epilepsy has been recognized as a priority for prevention and treatment among neurological and mental diseases by the World Health Organization.

Valproic acid (VPA) serves as a primary antiepileptic medication utilized in pediatric clinical use, exhibiting therapeutic efficacy across various types of seizures (Pong et al., 2023). Nevertheless, therapeutic index of VPA is narrow, and it can provoke a range of adverse reactions. It is recommended that the effective and safe treatment concentration range is 50–100 mg/L by the guidelines (Hiemke et al., 2018). VPA has high binding affinity to plasma protein, and the free fraction responsible for its pharmacological and toxic effects accounts for 5%–10% of the total drug. In order to reduce the risk of adverse effects and drug interactions, patients need therapeutic drug monitoring (TDM) to assess VPA levels especially for the children who usually has relatively lower plasma protein levels than adults (Charlier et al., 2021). Existing studies have established that VPA undergoes primary metabolism by hepatic enzymes in the liver and is subsequently eliminated through renal excretion after conjugation with glucuronic acid (Chatzistefanidis et al., 2012). However, due to the incomplete maturation of the hepatic enzyme system in children compared to adults, long-term use of VPA can negatively impact liver function. Additionally, dyslipidemia and significant weight gain are adverse effects associated with VPA that warrant attention. Lipids play diverse roles in cellular function, and abnormal plasma lipoprotein levels have been linked to the progression of certain diseases, such as atherosclerotic vascular disease (Abd Alamir et al., 2018) and nonalcoholic fatty liver disease (NAFLD) (Katsiki et al., 2016).

VPA, acting as a branched-chain short-chain fatty acid analogue, has been implicated in promoting fat storage, consequently contributing to weight gain. This effect occurs through mechanisms such as the reduction of leptin levels, decreased insulin sensitivity, and impairment of fatty acid β-oxidation. Indeed, VPA has been shown to elevate the level of leptin and exert dual actions. These actions involve modulation of the central nervous system to regulate appetite and energy expenditure, while simultaneously influencing the peripheral regulatory system to enhance insulin sensitivity and promote fatty acid oxidation, thereby reducing levels of circulating peripheral fatty acids. These effects contributed to the development of dyslipidemia and significant weight gain (Belcastro et al., 2013; Li et al., 2019). Dyslipidemia significantly heightens the risk of cardiovascular disease, and studies have suggested dyslipidemia in childhood is associated with an increased likelihood of cardiovascular events in adulthood. During childhood, atherosclerotic cardiovascular diseases are viewed as preclinical conditions, where dyslipidemia plays a crucial role. Fortunately, due to its impact on cardiovascular structure, dyslipidemia may be reversible during childhood. However, without proactive management, the condition may progress, ultimately leading to coronary heart disease, a primary cause of adult mortality (Arts et al., 2014; Pires et al., 2016; Yeung et al., 2021). According to data from the Chinese National Cardiovascular Center in 2010, cardiovascular diseases accounted for 40.4% of all deaths in China, making them the leading cause of mortality (Pan et al., 2016). Therefore, it is imperative to identify and manage dyslipidemia in children.

Currently, available studies on VPA-induced dyslipidemia (VID) in pediatric patients with epilepsy primarily concentrate on alterations in the lipid metabolism profile (Guo et al., 2022), potential mechanisms (Belcastro et al., 2013), and individual influencing factors (Attilakos et al., 2006). Long-term observational clinical studies reveal that VID is influenced not only by the drug properties but also by various physiological factors and individual differences. Identifying high-risk factors for VID in pediatric patients with epilepsy is beneficial for effective prevention strategies. Risk prediction models serve as valuable tools for this purpose (Wallisch et al., 2021; Canney et al., 2022). However, it is worth noting that there have been no reports of risk prediction models for dyslipidemia in patients on long-term VPA use, especially in pediatric patients, where both risk factors and models have not been documented.

Although VPA may induce dyslipidemia, the underlying factors contributing to this abnormality in children remain inadequately explored. This highlights the potential value of investigating VID within this specific pediatric cohort. Therefore, our retrospective cohort study was conducted to investigate the relationship between VID in pediatric patients and various physiological factors, medication history, and baseline biochemical indicators. Binary logistic regression was used to establish a dyslipidemia prediction model and identify independent risk factors. The primary objective of this study is to anticipate the likelihood of future dyslipidemia events in pediatric patients by monitoring their risk factors for emerging metabolic disorders. Furthermore, it aims to provide a more scientifically tailored approach to the clinical use of VPA, offering individualized medication recommendations and improving the precision of VPA treatment in children.

## 2 Materials and methods

### 2.1 Data source and collection

The data for this study were extracted from medical records and laboratory information systems of the Second Xiangya Hospital of Central South University, Hunan, China. We retrospectively collected relevant information regarding pediatric epilepsy patients who were admitted as inpatients and received VPA treatment, with concurrent monitoring of plasma concentrations. The data collection spanned a period from March 2018 to March 2023, encompassing a 5-year period. We also identified the potential risk factors associated with VID in pediatric patients with epilepsy. The following information was collected:

(1) Demographic parameters like patient ID, age, gender, BMI and so on;
(2) Medication information like VPA prescription, VPA C_trough_ and concurrent use of other antiepileptic drugs;
(3) Biochemical parameters like liver and kidney function parameters and blood routine parameters before VPA treatment, and lipid parameters after VPA treatment;
(4) Other information like past medical history, family history and previous medication.

All data were anonymized to ensure privacy protection for all participants. This study (Chinese Clinical Trial Registry: ChiCTR2300077071) was approved by the Medical Ethics Committee of the Second Xiangya Hospital of the Central South University, and all protocols were performed under the guidelines of the Helsinki Declaration (World Medical Association, 2013).

### 2.2 Inclusion and exclusion criteria

The inclusion criteria for this study were as follows: (1) aged younger than 16 years; (2) confirmed diagnosis of epilepsy, adhering to the diagnostic criteria outlined in the 2017 classification of the International League Against Epilepsy and Epilepsy syndrome (Riney et al., 2022); and (3) being a natural eating state. The exclusion criteria were as follows: (1) pre-existing severe conditions such as tumors, liver or kidney diseases, and endocrine disorders prior to initiation of VPA; (2) concurrent use of medications known to interfere with the endocrine system, such as growth hormones; (3) history of previous drug administration affecting lipid metabolism or documented abnormalities in lipid metabolism; (4) incomplete data pertaining to VPA trough concentration, basic clinical characteristics, and biochemical indicators in children; and (5) smoking or alcohol consumption.

### 2.3 Criteria for the evaluation of dyslipidemia

Based on the blood lipid levels subsequent to VPA treatment, children with epilepsy were stratified into two groups: the normal lipid group and the dyslipidemia group. The diagnostic criteria for dyslipidemia were established in accordance with the guidelines provided by the National Cholesterol Education Program (NCEP) in 2012 (Bamba, 2014; Burlutskaya et al., 2021). Dyslipidemia was diagnosed if any of the following four criteria were met under standard dietary conditions: (1) total cholesterol (TC) ≥ 5.17 mmol/L (200 mg/dL); (2) low-density lipoprotein cholesterol (LDL-C) ≥ 3.36 mmol/L (130 mg/dL); (3) high-density lipoprotein cholesterol (HDL-C) <1.03 mmol/L was defined as the low level (40 mg/dL); and (4) the cut-off value of triglycerides (TG) was ≥1.12 mmol/L (100 mg/dL) in children aged 9 years and younger, and ≥1.46 mmol/L (130 mg/dL) in children aged 10 years and older.

### 2.4 Statistical analysis

All statistical analyses were performed with SPSS (version 27, IBM), and the figures of analysis were presented by GraphPad Prism (version 8.0.1). Quantitative data were presented as mean ± standard deviation or median (interquartile range). The normality of quantitative data was tested using the Shapiro–Wilk test. Based on the normality results, either a *t*-test or non-parametric test was chosen to identify factors associated with statistically significant dyslipidemia events. Categorical data were described using percentages (N%) based on percentile calculations. Simultaneously, categorical data were analyzed using the crosstab χ2 test or fisher exact test depending on varying conditions. According to the type of data distribution, Pearson/Spearman rank correlation analyses were performed to explore the relationships between the selected factors and lipid parameters. Subsequently, clinically significant variables, and factors with statistical differences (*p* < 0.05) were included in the multivariate binary logistic regression analysis model (backward stepwise regression method) to determine the independent risk factors for dyslipidemia induced by VPA in pediatric patients with epilepsy. The results were evaluated by OR value and 95% confidence interval. The Hosmer-Lemeshow test and the area under the receiver operating characteristic (ROC) curve (AUC) were used to determine the predictive performance, and the maximum Youden index was used as the best cut-off value of the model. A two-tailed test with *p* < 0.05 was considered statistically significant.

## 3 Results

### 3.1 Study population and clinical characteristics

**FIGURE 1:**
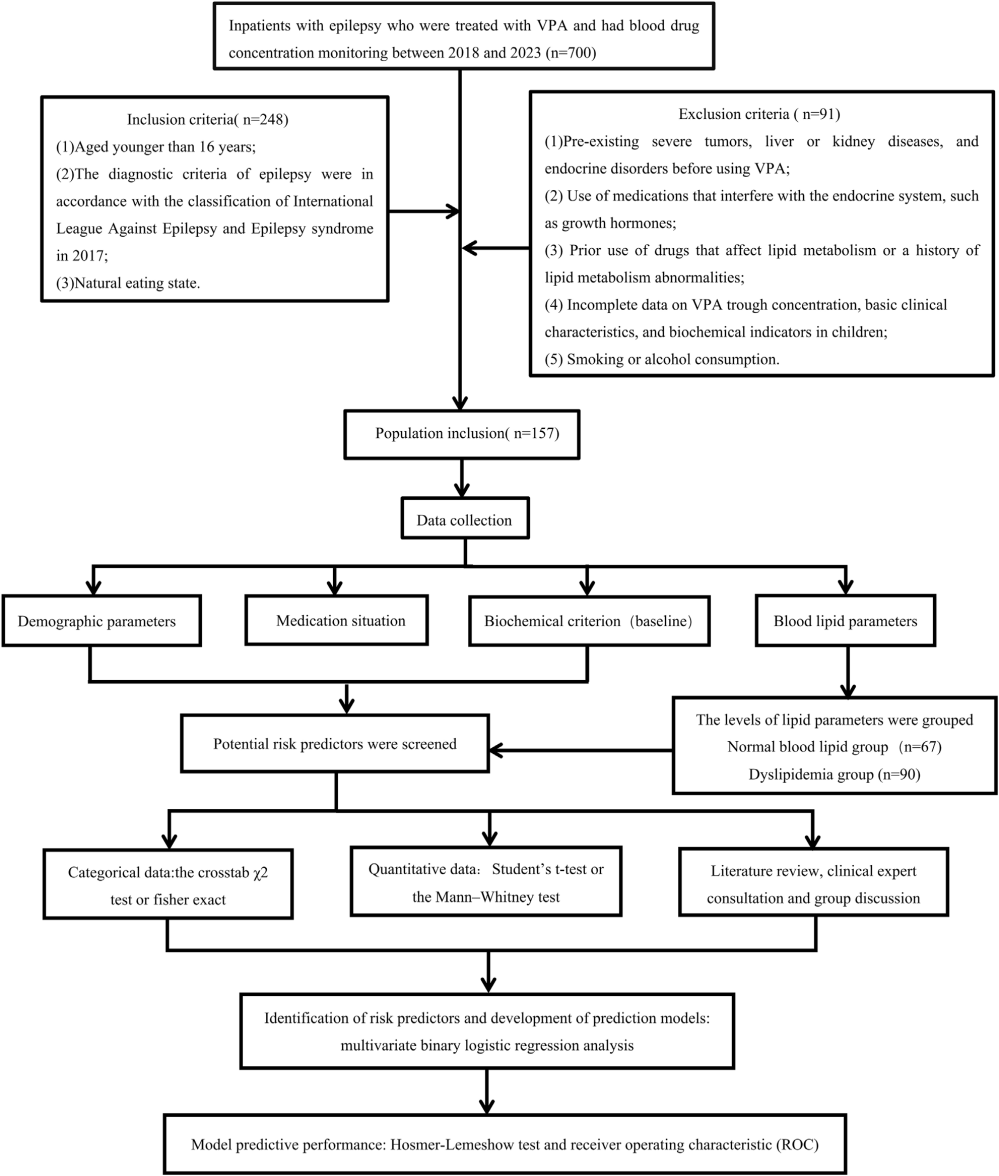
Participant flowchart and the research design.

We enrolled a total of 157 eligible patients between 2018 and 2023. The research design was illustrated in following flowchart (Figure 1). The demographic and main physiological indicators were summarized in Table 1. Among them, a total of 90 (57.32%) pediatric patients experienced dyslipidemia events. Additionally, within these 90 patients, 19 patients had high TC, 58 patients had high TG, 36 patients had low HDL-C, and 14 patients had high LDL-C. Further analysis revealed that among the pediatric patients with dyslipidemia, there were 35 cases (38.89%) with isolated high TG dyslipidemia, 24 cases (26.67%) with isolated low HDL-C dyslipidemia, 2 cases (2.22%) with isolated high LDL-C dyslipidemia, and the remaining 29 cases (32.22%) had mixed-type dyslipidemia.

**TABLE 1:** Patient characteristics in normal and dyslipidemia cohorts.

Parameters	Normal (n = 67)	Dyslipidemia (n = 90)	*p*-Value
Demographic variable			
Gender (male)	38 (56.71%)	63 (70.00%)	0.086
Age (year)	6.00 (3.00–9.00)	5.00 (2.00–11.25)	0.994
Weight (kg)	21.00 (13.00–32.00)	19.50 (13.88–32.00)	0.766
Height (cm)	116.00 (93.00–134.00)	114.00 (92.75–138.25)	0.886
BMI	15.97 (13.78–18.44)	17.11 (15.00–19.85)	0.038∗
Medication situation			
Standard daily dose (mg/kg)	20.57 ± 6.28	21.24 ± 6.99	0.536
VPA C_trough_ (ug/L)	58.82 ± 22.44	56.09 ± 22.08	0.447
Duration of medication			
Duration of medication≤3months	19 (28.4%)	52 (57.8%)	*p < 0.0001*∗
Duration of medication>3months	48 (71.6%)	38 (42.2%)	
Dosage form			
Oral liquid	44 (65.7%)	51 (56.7%)	0.511
Ordinary tablet	4 (6.0%)	6 (6.7%)	
Sustained release tablet	19 (28.4%)	33 (36.7%)	
Concomitant drug use (yes)			
Levetiracetam	25 (37.5%)	20 (22.2%)	0.039^∗^
Oxcarbazepine	7 (10.4%)	7 (7.8%)	0.562
Clonazepam	3 (4.5%)	11 (12.2%)	0.092
Lamotrigine	4 (6.0%)	4 (4.4%)	0.667
Topiramate	3 (4.5%)	10 (11.1%)	0.136
Biochemical criterion (baseline)			
WBC (10⁹/L)	7.34 (6.19–10.08)	7.81 (6.10–10.96)	0.743
HGB (g/L)	121.79 ± 10.10	117.02 ± 15.31	0.020^∗^
RBC (10^12^/L)	4.20 ± 0.41	4.22 ± 0.59	0.855
PLT (10⁹/L)	271 (210–328)	261.5 (198.75–347.75)	0.626
ALT (u/L)	14.30 (10.50–22.2)	15.20 (10.60–19.83)	0.949
AST (u/L)	28.40 (24–33.7)	30.85 (24.38–36.43)	0.274
TP (g/L)	64.40 (61.80–68.10)	64.60 (60.40–69.50)	0.999
ALB (g/L)	40.80 (38.60–44.00)	39.20 (36.03–42.68)	0.025∗
TBIL (umol/L)	5.20 (3.90–7.10)	3.65 (2.90–5.63)	*p < 0.0001∗*
DBIL (umol/L)	1.90 (1.50–2.40)	1.40 (0.90–1.90)	*p < 0.0001∗*
GLU (mmol/L)	4.56 (4.14–4.91)	4.77 (4.15–5.30)	0.217
Crea (umol/L)	28.90 (24.50–37.80)	32.35 (24.48–41.25)	0.174
UA (umol/L)	259.50 (213.90–298.20)	245.40 (192.55–319.13)	0.508

The value was presented as N (%), median (interquartile range) or mean ± standard deviation. C_trough_, trough concentration; ALT, alanine aminotransferase; TBIL, total bilirubin; TP, total protein; Crea, creatinine; UA, uric acid; GLU, blood glucose; WBC, white blood cells; HGB, hemoglobin; RBC, red blood cells; PLT, platelets.

The distinction was statistically significant, at the level of 0.05 (double tail).

In the comparison between the normal subgroup and the dyslipidemia subgroup, we observed that there were no statistically significant differences between the two groups in terms of gender, age, height, weight, standard daily dose, VPA C_trough_, dosage form, and other factors (Table 1). However, there were statistically significant differences between the two groups of children concerning BMI, the duration of medication, levetiracetam use, serum albumin (ALB), total bilirubin (TBIL), direct bilirubin (DBIL), and hemoglobin (HGB).

The correlation analyses between bilirubin (TBIL and DBIL) and lipid indexes (TG, TC, HDL-C, and LDL-C) have been carried out in Supplementary Table S1. It was found that TC, TG and LDL-C were negatively correlated with DBIL (TC: r = −0.221, *p* = 0.005; TG: r = −0.260, *p* = 0.001; LDL-C: r = −0.240, *p* = 0.002), and TG was negatively correlated with TBIL (r = −0.184, *p* = 0.021) as well. By contrast, HDL-C was positively correlated with TBIL (r = 0.233, *p* = 0.003).

### 3.2 Identification of independent risk factors and establishment of risk prediction models

Based on the results presented in Table 1, seven factors with statistically significant differences (*p* < 0.05) were identified: BMI, duration of medication, levetiracetam use, HGB, ALB, TBIL, and DBIL. Furthermore, seven additional potential risk factors, although showing no statistically significant differences in Table 1, were selected based on clinical advice, prior literature reviews (Tokgoz et al., 2012), and group discussions. These additional factors included VPA C_trough_, GLU, ALT, AST, WBC, gender, and clonazepam use. In total, we included 14 possible risk factors in the multifactorial binary logistic regression analysis model (using backward stepwise regression) to identify the independent risk factors associated with lipid abnormalities induced by VPA in children with epilepsy.

**FIGURE 2:**
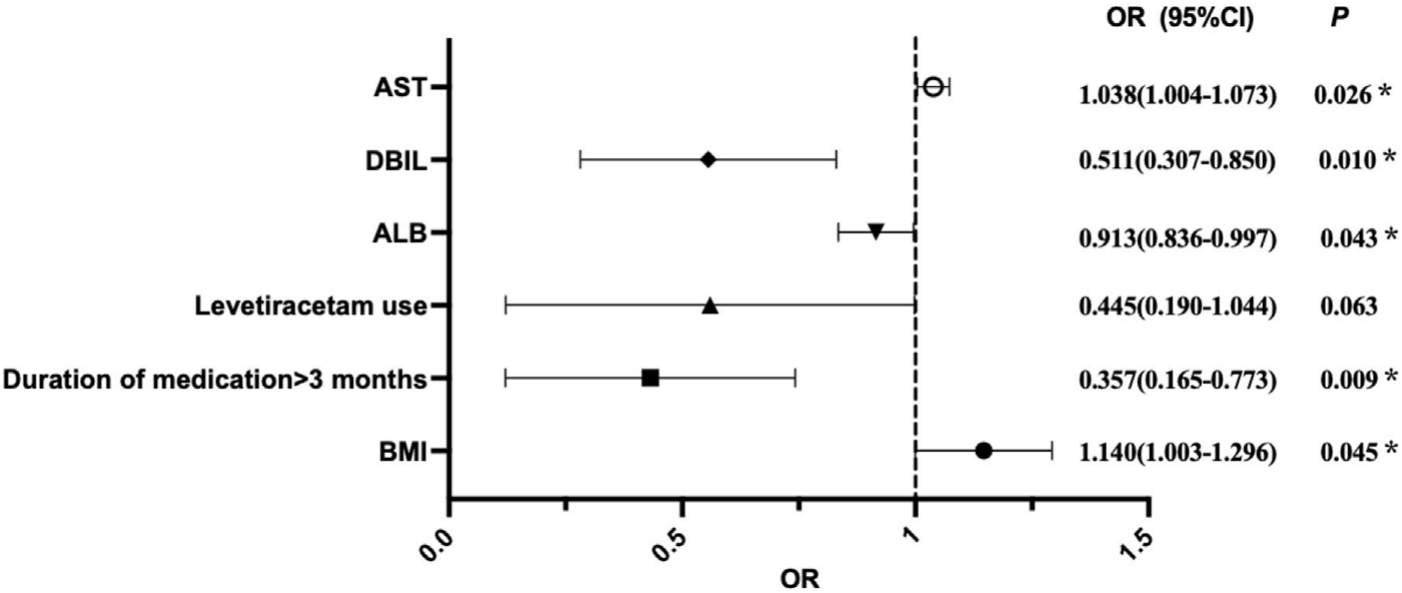
Visual forest plot of OR values for predictors of VID in pediatric patients with epilepsy by binary logistic regression analysis. OR = 1, indicating that the factor has no effect on the occurrence of VID; OR > 1, indicating that the factor is a risk factor; OR < 1, indicating that the factor is protective.

The results from binary logistic regression analysis, as well as the forest plot for odds ratios (Table 2; Figure 2) showed that BMI was an independent risk factor for VID in children with epilepsy (OR = 1.140, 95%CI:1.003–1.296, *p* = 0.045), suggesting that for every 1 kg/m^2^ increased in BMI, the risk of dyslipidemia in children with epilepsy increased by 1.140-fold. At the same time, AST was also determined to be an independent risk factor for dyslipidemia in children with epilepsy caused by VPA (OR = 1.038, 95% CI: 1.004–1.073, *p* = 0.026), suggesting that if AST increased by 1u/L, the risk of dyslipidemia in children with epilepsy would increase by 1.038-fold. On the contrary, a medication duration of more than 3 months was identified as an independent protective factor for VID in children with epilepsy (OR = 0.357,95% CI: 0.165–0.773, *p* = 0.009). Compared with the duration of medication less than 3 months, children with epilepsy who had been on medication for more than 3 months had a lower likelihood of developing dyslipidemia. ALB (OR = 0.913,95% CI: 0.836–0.997, *p* = 0.043) and DBIL (OR = 0.511,95% CI: 0.307–0.850, *p* = 0.01) were also independent protective factors of dyslipidemia in children with epilepsy caused by VPA. Within a certain range, high levels of ALB and DBIL can reduce the risk of dyslipidemia in these children. Additionally, levetiracetam use, clonazepam use, VPA C_trough_, gender, WBC, HGB, TBIL, GLU, ALT and other nine other factors had no significant effect on the occurrence of dyslipidemia.

**TABLE 2:** Binary logistic regression analysis of dyslipidemia events predictors.

Parameter	B	SE	Wald	df	*p*-Value	OR	95% CI
BMI	0.131	0.066	4.007	1	0.045*∗*	1.140	1.003–1.296
Duration of medication>3months	−1.03	0.394	6.828	1	0.009*∗*	0.357	0.165–0.773
Levetiracetam use	−0.81	0.435	3.465	1	0.063	0.445	0.190–1.044
ALB	−0.091	0.045	4.081	1	0.043*∗*	0.913	0.836–0.997
DBIL	−0.672	0.260	6.682	1	0.010*∗*	0.511	0.307–0.850
AST	0.037	0.017	4.927	1	0.026*∗*	1.038	1.004–1.073
Constant value	2.546	1.985	1.644	1	0.200	12.751	
Hosmer–Lemeshow test					0.471		

CI, confidence interval.

The variables was significant, at the level of 0.05 (double tail).

Hosmer–Lemeshow test; *p* > 0.05, indicating that the model fits well and statistic significantly.

The independent influencing factors obtained in the binary logistic regression were combined to form the subsequent joint predictive factors. According to the results of logistic regression (Table 2), an equation for the combined predictive factor was established:

Logit P=2.546+0.131×BMI−1.03×duration of medication×A−0.091×ALB−0.672×DBIL+0.037×AST

(“A = 1” if duration of medication>3months, “A = 0” duration of medication ≤ 3months”)

### 3.3 Assessment of the clinical prediction model’s performance and accuracy

The Hosmer-Lemeshow goodness of fit test was a valuable tool to assess the calibration of a predictive model, indicating how well the predicted values align with observed values (Table 3). In this study, the test result (*p =* 0.471) indicated that the difference between the predicted value and the observed value was not statistically significant when compared to a significance level of 0.05. This suggested that the predicted results of this model were in good agreement with the actual incidence of VID in children with epilepsy, which was scientific and practical.

**TABLE 3:** The predictive efficacy of each risk predictor for dyslipidemia.

Parameter	AUC	*p*-Value	95% CI	Sensitivity	Specificity	Youden index	Cutoff value
Combined prediction	0.777	<0.0001	0.706–0.849	0.733	0.746	0.479	0.558
DBIL	0.690	<0.0001	0.607–0.772	0.578	0.731	0.309	1.55
Duration of medication	0.647	0.002	0.560–0.734	-	-	-	-
ALB	0.604	0.025	0.516–0.692	0.356	0.836	0.192	37.75
BMI	0.597	0.038	0.507–0.687	0.722	0.478	0.2	15.35
AST	0.551	0.274	0.461–0.641	0.489	0.701	0.19	31.65

Combined prediction, indicating that a combined forecast of five independent predictors:DBIL, duration of medication, ALB, BMI, and AST.

**FIGURE 3:**
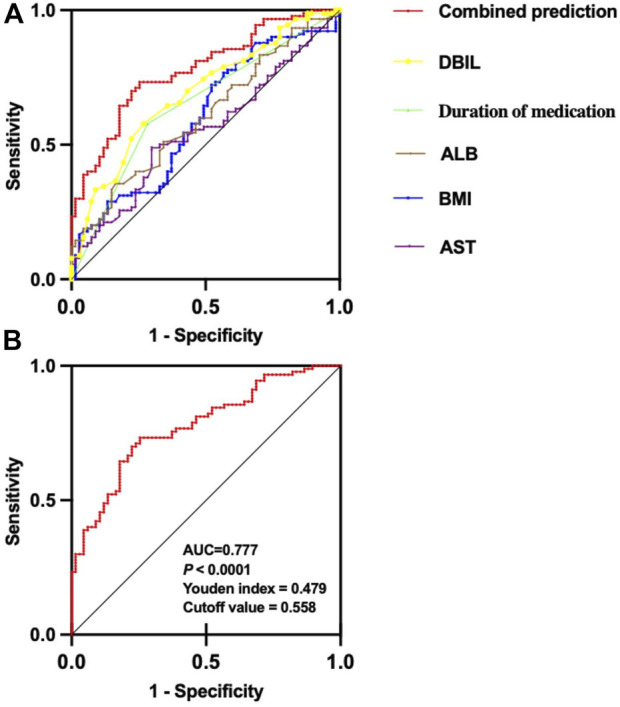
ROC curve of each risk predictor and combined predictor of VID in pediatric patients with epilepsy. **(A)** ROC curve comparison of each risk predictor and combined predictor of dyslipidemia; **(B)** ROC curve of combined prediction.

The receiver operating characteristic curves (ROC) of each independent risk factor and combined predictive factor were drawn to determine the predictive power of each factor. The results (Table 3; Figure 3) showed that the predictive capability of DBIL (AUC = 0.690, 95% CI: 0.607–0.772, *p* < 0.0001) was stronger than that of other factors alone. Furthermore, the combined predictive capability of the five independent risk factors was stronger than the independent prediction ability of each factor. The AUC of combined prediction was 0.777 (95% CI: 0.706–0.849, *p* < 0.0001), the optimal cutoff value was 0.558, the Youden index was 0.479, the sensitivity was 0.733, and specificity was 0.746 (Figure 3).

In summary, the combined prediction model consisting of DBIL, Duration of medication, ALB, BMI, and AST demonstrated excellent predictive capabilities.

## 4 Discussion

Currently, there exists a notable research gap regarding the identification of risk factors for dyslipidemia in pediatric epilepsy patients undergoing VPA treatment. In this study, we conducted a retrospective analysis of the lipid profiles of 157 pediatric epilepsy patients undergoing VPA treatment to discern independent risk factors for dyslipidemia in this specific group of children. We confirmed that: (1) DBIL, Duration of medication, and ALB emerged as independent protective predictors against dyslipidemia induced by VPA in pediatric epilepsy patients, while BMI and AST were risk factors; (2) among the individual risk factors, DBIL had a stronger predictive ability compared to other factors when predicting individually; and (3) the combination of these five risk factors exhibited the strongest predictive capability compared to individual risk factors.

Our study underscores the significance of DBIL levels prior to VPA treatment as a prominent predictor of VID in pediatric patients with epilepsy. Compared with other single predictors, DBIL demonstrated the strongest predictive power, with an area under the ROC curve of 0.690 (95%CI: 0.607–0.772, *p* < 0.0001). Therefore, within the normal range, high DBIL was a protective factor for blood lipids in children with epilepsy treated with VPA. This was confirmed by the correlation between DBIL and blood lipid indexes. Bilirubin, a product of heme metabolism, arises from the breakdown of iron porphyrin compounds within aging red blood cells. This process yields two main forms of bilirubin: conjugated bilirubin (DBIL) and unconjugated bilirubin (indirect bilirubin). It serves a clinical diagnosis of hemolysis, neovascularization and hepatobiliary diseases (Fevery, 2008). VPA undergoes intricate metabolic transformations primarily in the liver. Notably, its reactive metabolites have been reported to be associated with hepatotoxicity (Guo et al., 2019). Abnormal elevation of bilirubin is one manifestation of impaired liver function. In an individualized treatment model for patients with bipolar disorder receiving VPA, researchers observed that indirect bilirubin was positively correlated with VPA daily dose (Zheng et al., 2022). Interestingly, it is indicated that bilirubin possesses potent antioxidant properties, which include anti-inflammatory, antioxidant, and free radical scavenging properties (Stocker et al., 1987). The elevation of bilirubin facilitates the breakdown and metabolism of low-density lipoprotein cholesterol, leading to a reduction in its concentration in the body and consequently exerting an anti-atherosclerotic effect (Vogel et al., 2017). Therefore, a decrease in serum bilirubin concentration weakens its antioxidant efficacy, potentially resulting in abnormal lipid metabolism. Furthermore, animal experiments have demonstrated that bilirubin can regulate genes involved in lipid metabolism by activating peroxisome proliferator-activated receptor alpha (PPAR-α), thus contributing to lipid regulation (Stec et al., 2016). A cross-sectional and longitudinal study conducted by Eiji Oda, involving a health screening population, aimed to explore the relationship between serum bilirubin levels and dyslipidemia. The study revealed a significant correlation, indicating a decrease in total bilirubin levels was notably associated with hypertriglyceridemia, low HDL and hypercholesterolemia (Oda, 2015). This was consistent with the findings of our study. Dyslipidemia serves as a crucial indicator of metabolic syndrome (MS). The relationship between different bilirubin parameters and MS was evaluated in a Korean study, which found that direct bilirubin, indirect bilirubin, and total bilirubin levels were negatively correlated with MS, but DBIL was more closely related to MS (Jo et al., 2011). Therefore, within the normal range, DBIL was a protective factor for VID in children with epilepsy.

Interestingly, our study also revealed that pediatric epilepsy patients undergoing VPA treatment for more than 3 months exhibited a protective effect against VID compared to those with a medication duration of less than 3 months. A study by Kusumastuti K, employing logistic regression, made a similar discovery. Specifically, it was found that the risk of developing high total cholesterol levels in adult patients with VPA use for more than 12 months was 3.68 times lower than that with VPA use for less than 12 months (Kusumastuti and Jaeri, 2020). In a meta-analysis conducted in 2022, researchers observed that long-term VPA treatment had the potential to reduce total cholesterol and low-density lipoprotein cholesterol levels. This effect may be achieved by enhancing certain organs/cell organelles that contribute to improving cholesterol metabolism (Guo et al., 2022). Huang et al. demonstrated that VPA exhibits the capability to mitigate oxidative stress in the endoplasmic reticulum via the pathway of glycogen synthase kinase 3/β which contributes to cholesterol metabolism and the pathogenesis of atherosclerosis (Huang et al., 2017). On the other hand, the main pathway of VPA bioconversion involves glucuronidation, and glucuronidase may be inhibited by VPA or its metabolites, resulting in decreased synthesis of TC, LDL-C and HDL-C. On the contrary, children with VPA have been reported to develop hyperinsulinemia, which can stimulate lipogenesis, resulting in the accumulation of triglycerides and hypercholesterolemia (Kanemura et al., 2012; Belcastro et al., 2013). This suggests that the mechanism of action and metabolic effects of VPA *in vivo* are very complex. VPA may exert bidirectional effects on lipid metabolism through different mechanisms. In the early stage of VPA treatment, the insulin level responds rapidly, leading to an abnormal trend of blood lipid elevation. However, with the long-term use of VPA, VPA acts on other pathways to reduce blood lipid levels. Similar trends were observed in both adults and children. However, due to the immature development of liver and other metabolic tissues in children, there were differences in the time points of the bidirectional regulation of blood lipids by VPA. Weight gain is recognized as one of the most overt features of dyslipidemia, and according to the literature, weight gain during VPA treatment can be observed within the first 3 months of treatment (Verrotti et al., 2011; Sidhu et al., 2017). Furthermore, another study indicated that VPA administration reduced blood glucose levels and increased motivation to eat, potentially leading to increased energy intake, particularly in the early stages of treatment (Martin et al., 2009). At the same time, due to the immature but fast development of liver and other organs in children, they may respond more rapidly and severely to drug effects. Therefore, we categorized treatment duration as more or less than 3 months, and found that close attention should be paid to blood lipid indexes in children with epilepsy treated with VPA in the early stage.

Baseline ALB was also identified as a protective predictor of VID in children with epilepsy. ALB is the predominant protein in human plasma and represents the most abundant circulating protein in the extracellular compartment, which plays a critical role in the distribution of body fluids (Caraceni et al., 2013). Its strong binding and transport functions enable it to serve as a ligand for various drugs, thereby influencing the pharmacokinetics of numerous medications. Additionally, ALB engages in binding with bilirubin, free fatty acids, minerals, and hormones, participating in multiple metabolic reactions (Levitt and Levitt, 2016; Chen et al., 2021). Studies have been shown that a deficiency in ALB disrupts intravascular lipolysis, resulting in a shortage of free fatty acids and a diminished clearance of triglyceride, consequently leading to hypertriglyceridemia (Figueira et al., 2010). Interestingly, research has demonstrated that ALB infusion helps alleviate dyslipidemia in models of anaplastic anemia, providing compelling evidence for the positive role of serum albumin in regulating lipoprotein metabolism (Rosipal et al., 2006; Del Ben et al., 2013; Crook, 2016). All of these observations align with the findings of our study, indicating that ALB serves as a protective predictor of dyslipidemia in children with epilepsy. Elevated levels of ALB before the administration of VPA are beneficial for the protection of lipids and reduction of dyslipidemic events in children with epilepsy.

BMI and AST before VPA treatment were considered independent risk predictors of VID in pediatric patients with epilepsy after accounting for the effects of other factors. BMI is a reliable indicator of obesity, which is an important symptom of dyslipidemia, as supported by numerous studies (Zaid et al., 2017; Yu et al., 2022). Notably, one of the adverse effects of VPA is the potential increase in BMI and the risk of obesity in patients. Consequently, baseline BMI before VPA treatment serves as a crucial risk predictor for dyslipidemia in pediatric patients with epilepsy. Given the pivotal role of the liver in regulating lipid metabolism, it is evident that liver injury can significantly impact lipid metabolism. ALT and AST represent the two most common aminotransferase markers used clinically to assess liver injury or the severity of liver disease. The effect of VPA administration on the elevation of liver enzymes is evident, especially in young children with immature liver function. Oxidative stress in the mitochondria due to VPA usage can further exacerbate liver damage. Hence, it is imperative to regularly monitor liver function in patients with VPA, especially in pediatric patients, both prior to treatment and during its course, to prevent the risk of liver injury as well as lipid metabolism disorders.

We also explored VPA C_trough_ *in vivo* as an independent risk factor for VID in children with epilepsy. Notably, previous studies examining the risk factors for hyperammonemia induced by VPA revealed that the VPA C_trough_ and the standard daily dose of VPA could significantly predict the blood ammonia levels (Duman et al., 2019). Unfortunately, in this study, the VPA C_trough_ and the standard daily dose of VPA did not emerge as predictors of dyslipidemia in binary logistic regression. Further analysis was conducted to examine the relationships between standard daily dose of VPA, VPA C_trough_ and the four lipid parameters (TC, TG, HDL-C and LDL-C) in the whole patient cohort. However, no significant correlations were found (Supplementary Table S2). Specifically, in the dyslipidemia group, the associations between lipid parameters (TC, TG, HDL-C and LDL-C) and the standard daily dose of VPA, VPA C_trough_ were also investigated, and no results approached significance either (Supplementary Table S3). These findings suggested that VID in epileptic children in this study was no related to VPA C_trough_ or standard daily dose.

There were several limitations in this study. First, this study was carried out in a single center, and the study population primarily comprised children, resulting in a relatively small sample size. Therefore, the prediction model could not yet be extrapolated to other centers. To enhance the generalizability of findings, it is essential to conduct multi-center collaborative research to further expand the sample size. Second, it was a retrospective study, although various significant factors have been analyzed, there remains the possibility of uncontrollable interfering factors. Moreover, there could exist unknown or unmeasured risk factors that were not accounted for in this analysis. Subsequent prospective studies can be designed to incorporate more possible risk factors. Additionally, such studies could implement controls for changes during follow-up and undertake further systematic evaluations. Finally, due to the limited prevalence of genetic testing in clinical practice, we were unable to collect information about the patients themselves regarding the genotypic polymorphisms that were specific influences on VPA metabolism. It is found that the genotype polymorphisms of patients, such as CYP2C9 and CYP2A6, were important factors for dyslipidemia (Yoon et al., 2020). At the same time, pharmacogenetics is helpful to understand the treatment efficacy and adverse effects. It has been reported that the polymorphism of the SCN1A gene may play a role in the response to anti-epileptic drugs in patients with drug-resistant epilepsy, which is of great significance for clinical practice (Margari et al., 2018). Therefore, it is necessary to conduct prospective studies by combining multiple centers, expanding the sample size, and expanding important potential risk factors.

## 5 Conclusion

This study has identified DBIL, Duration of medication, ALB, BMI, and AST as independent risk factors for VID in pediatric epilepsy patients. By combining these five risk factors into a composite predictive factor, we can predict 77.7% of blood lipid abnormal events, demonstrating a strong predictive capability. This finding is highly significant for pediatric epilepsy patients undergoing VPA treatment. Also, these factors can guide precise VPA dosing, maximizing the therapeutic effects of VPA while minimizing the risk of blood lipid abnormalities. This approach also aims to reduce the risk of future cardiovascular events in children. Ultimately, it provides a more scientifically tailored approach to the clinical use of VPA, enhancing its precision in pediatric patients.

## Data Availability

The datasets presented in this article are not readily available because of privacy reasons. Requests to access the datasets should be directed to the corresponding author (hualincai@csu.edu.cn).
