# Supplementary material for: Pre-treatment risk predictors of valproic acid-induced dyslipidemia in pediatric patients with epilepsy
Source: Front Pharmacol. 2024 Apr 2;15:1349043. doi: 10.3389/fphar.2024.1349043 (PMC11018995; doi:10.3389/fphar.2024.1349043)
Supplement: Supplementary file 1 [file DataSheet1.PDF]

Supplementary Material

Pre-treatment risk predictors of valproic acid-induced dyslipidemia in pediatric patients with epilepsy

Tiantian Liang [1,2], Chenquan Lin [1,3], Hong Ning [2], Fuli Qin [4], Bikui Zhang [1,3,5], Yichang Zhao [1,3], Ting Cao [1,3], Shimeng Jiao [1,3], Hui Chen [1,3], Yifang He [1,3], Hualin Cai [1,3,5*] (hualincai@csu.edu.cn)

Supplementary Table 1. Correlation analyses between bilirubin and blood lipid parameters

Supplementary Table 2. Correlation analyses among the standard daily dose, VPA C_{trough} and blood lipid parameters in the whole cohort

Supplementary Table 3. Correlation analyses among the standard daily dose, VPA C_{trough} and blood lipid parameters in the dyslipidemia group

Table S1. Correlation analyses between bilirubin and blood lipid parameters

		TBIL	DBIL
TC	Coefficient Index (r)	0.053	-0.221*
	P	0.514	0.005
TG	r	-0.184*	-0.260*
	P	0.021	0.001
HDL-C	r	0.233*	-0.005
	P	0.003	0.954
LDL-C	r	0.007	-0.240*
	P	0.930	0.002

* The distinction was statistically significant, at the level of 0.05 (double tail).

Table S2. Correlation analyses among the standard daily dose, VPA C_{trough} and blood lipid parameters in the whole cohort

		the standard daily dose	VPA C_{trough}
TC	Coefficient Index (r)	-0.048	-0.108
	P	0.553	0.178
TG	r	-0.06	-0.05
	P	0.453	0.531
HDL-C	r	0.058	0.022
	P	0.47	0.785
LDL-C	r	-0.142	-0.152
	P	0.076	0.057

C_{trough}, trough concentration.

Table S3. Correlation analyses among the standard daily dose, VPA C_{trough} and blood lipid parameters in the dyslipidemia group

		the standard daily dose	VPA C_{trough}
TC	Coefficient Index (r)	-0.094	-0.092
	P	0.378	0.389
TG	r	-0.025	0.057
	P	0.816	0.593
HDL-C	r	-0.05	0.024
	P	0.643	0.826
LDL-C	r	-0.176	-0.131
	P	0.097	0.218

C_{trough}, trough concentration.
